# Haptic feedback helps bipedal coordination

**DOI:** 10.1007/s00221-016-4689-2

**Published:** 2016-06-04

**Authors:** Eefje G. J. Roelofsen, Jurjen Bosga, David A. Rosenbaum, Maria W. G. Nijhuis-van der Sanden, Wim Hullegie, Robert van Cingel, Ruud G. J. Meulenbroek

**Affiliations:** 1 Donders Centre for Cognition, Donders Institute for Brain, Cognition and Behaviour, Radboud University Nijmegen, P.O. Box 9104, 6500 HE Nijmegen, The Netherlands; 2 Research Group Musculoskeletal Rehabilitation, HAN University of Applied Sciences, P.O. Box 6960, 6503 GL Nijmegen, The Netherlands; 3 Department of Psychology, Pennsylvania State University, University Park, PA 16802 USA; 4 Scientific Institute for Quality of Healthcare, Radboud University Medical Center, P.O. Box 9101, 6500 HB Nijmegen, The Netherlands; 5 Practice for Physiotherapy Hullegie and Richter, Geessinkbrink 7, 7544 CW Enschede, The Netherlands; 6 Sport Medical Center Papendal, Papendallaan 7, 6816 VD Arnhem, The Netherlands

**Keywords:** Active-assisted motion, Cognition, Bipedal, Coordination, Haptic tracking, Passive movement, Physical therapy, Visual feedback

## Abstract

The present study investigated whether special haptic or visual feedback would facilitate the coordination of in-phase, cyclical feet movements of different amplitudes. Seventeen healthy participants sat with their feet on sliding panels that were moved externally over the same or different amplitudes. The participants were asked to generate simultaneous knee flexion–extension movements, or to let their feet be dragged, resulting in reference foot displacements of 150 mm and experimental foot displacements of 150, 120, or 90 mm. Four types of feedback were given: (1) special haptic feedback, involving actively following the motions of the sliders manipulated by two confederates, (2) haptic feedback resulting from passive motion, (3) veridical visual feedback, and (4) enhanced visual feedback. Both with respect to amplitude assimilation effects, correlations and standard deviation of relative phase, the results showed that enhanced visual feedback did not facilitate bipedal independence, but haptic feedback with active movement did. Implications of the findings for movement rehabilitation contexts are discussed.

## Introduction

Some inter-limb coordination patterns are more stable than others. In-phase movements of the two hands tend to be more stable than anti-phase hand movements, and anti-phase hand movements tend to be more stable than other phase relations (e.g., Amazeen et al. 1998; Cattaert et al. 1999; Heuer 1993; Ivry et al. 2004; Kelso 1984; Swinnen 2002). In addition, bimanual tasks that require each limb to move over a different distance are more difficult to produce than tasks in which the limbs move over equal distances. An assimilation effect occurs: The limb moving a shorter distance tends to overshoot the target, and the limb moving a longer distance tends to undershoot the target (e.g., Diedrichsen et al. 2001; Doumas et al. 2008; Heuer and Klein 2005; Peper et al. 2008; Sherwood 1994; Weigelt and Cardoso de Oliveira 2003).

One hypothesis about the source of this pattern of results is that it reflects cognitive limitations, i.e., patterns that are easier to plan are also easier to produce (e.g., Debaere et al. 2001, 2004; Wenderoth et al. 2005). Two methods have been devised to test this hypothesis. One is to simplify the visual feedback that is given about inter-limb coordination. The idea is that if actors have difficulty representing the actions they are supposed to produce, then eliminating that representational difficulty should eliminate the performance problem. In support of this logic, Mechsner et al. (2001) showed that participants could perform complex bimanual movement patterns when the visual feedback given about the actions obviated complex movement planning. In their experiment, participants were instructed to turn two visible flags in an in-phase or anti-phase relationship by means of two cranks hidden under a table. To produce the in-phase coordination pattern, participants had to produce a 4:3 bimanual polyrhythm because the cranks were geared differently. After a short training period, participants were able to perform the 4:3 polyrhythm with great ease, something they could not do without the special visual feedback afforded by the geared flags (see also Boyles et al. 2012; Diedrichsen et al. 2001; Franz and McCormick 2010; Franz et al. 2001; Ivry et al. 2004; Kovacs et al. 2010; Kovacs and Shea 2010).

Another method to facilitate complex inter-limb coordination is to create a situation in which subjects can move their limbs in response to light touch on moving objects or via an external guide by maintaining light contact with the guide’s moving body. This method is called haptic tracking. An example of haptic tracking is holding hands with another person while walking together, or slow dancing with a partner. Rosenbaum et al. (2006) studied haptic tracking as a means of facilitating inter-limb coordination. They asked university students to keep their hands in contact with two objects moved by one or two human drivers. The participants followed the moving objects, which they could not see while performing the task. To prevent the possibility that the participants’ hands were passively dragged, the objects were decoupled (by means of a magnetic system) from the movement of the drivers when the participant exceeded a certain amount of pressure. In a series of experiments, Rosenbaum and colleagues showed that haptic tracking allows people to simultaneously track a circle with the one hand and a square with the other hand. In addition, participants could easily follow circular motions with 3:4 or 4:3 frequency ratios. Such patterns cannot be generated by healthy individuals without haptic or simplified visual feedback (e.g., Franz et al. 2001).

As suggested by Rosenbaum et al. (2006), haptic tracking may ‘bypass’ the cognitive system by eliminating the need to simultaneously plan two different motion shapes. Instead, it allows participants to respond to felt shear forces. If a shear force is felt on a hand, a motion can be instantly generated that nulls the error. The latency of the correction can be almost instantaneous owing to the directness of the stimulus–response mapping: A felt shear force in one direction can be immediately compensated for by a movement in that same direction. The direct stimulus–response mapping can be attributed to direct ipsilateral connections across the central sulcus between primary somatosensory cortex and primary motor cortex (Kelly and Dodd 1991).

In the present study, we asked whether the two kinds of feedback just described—enhanced visual feedback and haptic feedback resulting from haptic tracking—could also benefit bipedal coordination. We predicted it would because walking is strongly affected by cognitive tasks (Beurskens and Bock 2012, 2013; Li et al. 2012; Woollacott and Shumway-Cook 2002), though in these studies it was unclear whether the interference was motoric, visual, or both because subjects had to visually monitor their surroundings as they walked while counting backwards or holding lists in memory. We sought to address this issue aside but were also interested in bipedal haptic tracking for an applied reason. We wondered whether one or both of the feedback methods could provide a new therapeutic approach to address locomotion disabilities of leg or foot control. We comment on this matter further in Discussion section.

To pursue the issues of interest, we asked seated participants to make rhythmic movements of the left and right feet in the sagittal plane—in–out movements—either when the amplitudes of the foot movements were same or different. One foot per participant was supposed to generate the same amplitude in all conditions (the reference foot), whereas the other foot was supposed to generate different amplitudes in the various conditions (the experimental foot). We were interested in, among other things, the effects of the experimental foot on the reference foot. Our question was whether the reference foot would be able to move more independently of the experimental foot when haptic tracking or special visual feedback was used. We recorded the foot displacements digitally and recorded the horizontal (forward) and vertical (downward) forces with force plates. Four different conditions were tested. In the haptic passive (HP) condition, the subjects let their feet be dragged by sliders on which their feet rested; the sliders were moved by two independent human drivers. In the haptic tracking (HT) condition, the subjects moved their feet based on light touch and the same two independent human drivers moved the sliders. In this condition, subjects were instructed to move along with the movement of the sliders, whereas in the HP condition the subjects were explicitly told to keep the legs relaxed and let the feet be dragged by the sliders. In the visual veridical (VV) condition, the subjects generated their left and right foot movements, relying on veridical visual feedback while doing so. Here only the subjects drove the sliders. Finally, in the visual-enhanced (VE) condition, the subjects generated the left and right foot movements themselves, as in the VV condition, but we manipulated the visual feedback by adding a gain factor to the feedback of one of the feet. By doing so, the visual feedback was presented as if equal amplitudes were required in every condition, while in fact in two of three conditions the participant had to generate amplitude differences with the feet in order to successfully reach the targets on the screen.

We hypothesized that the reference foot’s amplitude would be strongly affected by the experimental foot’s amplitude in the visual veridical (VV) condition and less affected in the visual-enhanced (VE) condition. This prediction mirrored the one made by Mechsner et al. (2001). Moreover, to the extent that planning of the two feet’s motions is required in both visual conditions but not in the HT condition, we hypothesized that the reference foot’s amplitude would be less affected by the experimental foot’s amplitude in the haptic tracking (HT) condition than in the visual (VV and VE) conditions as evidenced by smaller assimilation as regards foot amplitudes and also by lower correlations and larger standard deviations of the relative phase between the sagittal-plane foot displacement functions.

A second prediction was that the phasic downward forces of the feet on the sliders would be significantly higher in the HT condition than in the HP condition, based on the idea that in bipedal haptic tracking the guided movement is realized by an active, phasic contribution to the generation of the feet’s movements.

The third and final prediction concerned the horizontal forces between the sliding foot panels and driver handles (in the in–out direction). We expected the horizontal, phasic forces to be higher in the HP condition rather than in the HT condition. In the HT condition, we asked participants to actively contribute to the movements, so drivers and participants both exerted forces on the panels in the same direction, i.e., the foot panels went backwards (in) and forwards (out), whereas in the HP condition the drivers had to drag the ‘dead weight’ of the participants leg back and forth. The latter was expected to result in higher phasic forces in the horizontal direction.

## Methods

### Participants

Seventeen healthy young adults (14 females and 3 males) with a mean age of 23 years (SD 3 years) participated in this study. All participants were right-footed as determined by a Dutch version of the Waterloo Footedness Questionnaire (Elias et al. 1998). No participant reported injuries of the lower extremities or spine. Leg length was determined by measuring the distance (in cm) between the anterior superior iliac spine (ASIS) and the medial malleolus of the ankle. This measure was used to estimate the appropriate chair position and height for each participant. For each experiment, two additional participants served as drivers. All participants and the drivers provided written informed consent. The study was conducted in accordance with the Helsinki Declaration and approval by the Radboud University Ethics Committee.

### Apparatus

**Fig. 1:**
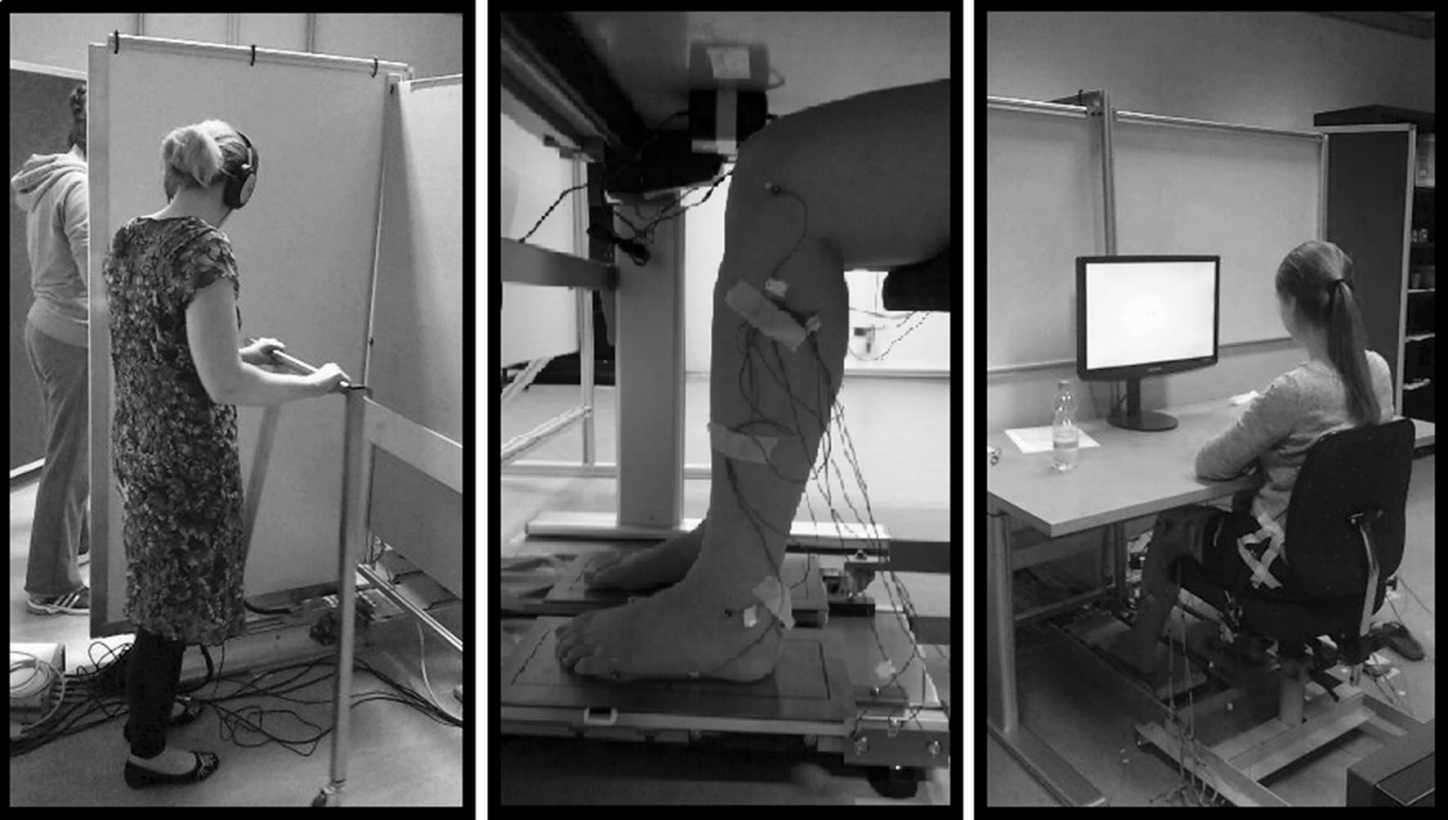
Experimental setup. *Left panel* The two drivers, shielded from each other and from the participant, held the handles (visible only for the nearer driver) and moved them in time with high and low tones, which they heard over headphones. *Middle panel* Close-up of the feet on the sliding foot supports. *Right panel* A participant seated in the chair with her feet on the sliding foot supports

The heights of the chair and table at which the participant sat were adjusted to maximize each participant’s comfort and to prevent sight of the legs (see Fig. 1). Also, the knee angle of the participants with their feet placed on a marked center on the sliding supports was 90°, as determined with a goniometer. The participant faced a computer screen that provided experimental instructions as well as online visual feedback about task performance. Brakes prevented the chair from moving, except before and after the experiment when the participant was moved into or out of position. Participants wore shorts and removed their shoes and socks so their bare feet rested on the two aluminum sliders. These were mounted on linear guides that allowed for motion in the sagittal plane. The sliders were mechanically coupled with driver handles that were manipulated by two confederates (the ‘drivers’). Hardboard partitions separated the drivers from the participant and from each other. Drivers wore headphones and received computer-generated auditory pacing sounds, indicating the target movement frequency of 2.0 Hz and the direction (forward or backward) of the movements with the driver handles. A low tone instructed the driver to push the handle forward, and a high tone instructed the driver to pull the handle back, resulting, ideally, in the generation of 1 motion-cycle per second (1.0 Hz). The frequency of the feet displacements was based on studies regarding human leg swinging in locomotion (Holt et al. 1990; Wagenaar and van Emmerik 2000).

The amplitudes of the movements that the drivers were supposed to produce were represented by two colored lines on a ruler next to the guiding system that the drivers looked at on the floor. The amplitude of 120 mm was indicated by two red lines, the amplitude of 90 mm was indicated by two blue lines, and the amplitude of 60 mm was indicated by two green lines. Prior to each trial, the two drivers were told, via headphones, what amplitude to produce and what the associated color guidelines were.

The sliding foot supports contained two button load cells of type 31370 (0–200 kg, CZL204E, Phidgets Inc., Calgary, Canada) integrated in the center, measuring vertical forces exerted by the left foot on the left foot support and by the right foot on the right foot support. The precision of the button load cells was 0.02 % F.S. Two S-shaped load cells of type 31380 (0–100 kg, CZL301C, Phidgets Inc., Calgary, Canada), positioned between driving handles and foot supports, were used to record the horizontal forces, i.e., the push–pull forces of the drivers–participant system. The precision of the S-shaped load cells was 0.2 % F.S. The sensitivity of the four cells was chosen to prevent the possibility of damaging the sensor permanently by applying a load that would exceed 120 % for both types. The offsets were adjusted for each sensor individually.

Two infrared-emitting diodes (IREDS) were attached to the lateral malleoli of the participants’ ankles. Two IREDS were also attached to the sliding foot supports. Translations of the IREDS were recorded at 100 Hz with a spatial accuracy of 0.2 mm with a 3D motion tracking system (OPTOTRAK 3020, Northern Digital Inc., Waterloo, Canada). Signals from the four load cells were amplified 6000 times by means of a ‘Strain Gauge Amplifier SC802B’ (TD2000042), designed by the Technical Support Group (TSG) of the Radboud University. The output of these amplified signals was connected to the analog inputs of the ODAU (Optotrak Data Acquisition Unit) of the Optotrak system. This enabled us to record the forces simultaneously with the recorded Optotrak movement data.

### Procedure and design

Participants were asked to make rhythmic in–out movements of the left and right feet, either in conditions where the amplitudes of the feet movements were the same or different. All of the left and right foot movements were in-phase. For a given participant, either the left foot or right foot was always supposed to cover a fixed amplitude of 150 mm. We called this the reference foot. For eight of the 17 subjects, the left foot served as the reference foot, while for the remaining subjects the right foot served as the reference foot. For every subject, the other foot, the experimental foot, was supposed to cover three different amplitudes—either the same amplitude (150 mm) as the reference foot, 30 mm less (120 mm), or 60 mm less (90 mm).

Each subject performed each combination of left–right foot amplitudes in four different conditions that were presented in a random order per subject, with the order of the experimental foot amplitudes being random per participant within each condition. Each condition–amplitude combination was tested in 10 trials. A short break of approximately 3 min was introduced after finishing each condition. The three amplitude pairs within a condition were presented in a random order (without replacement) per participant. Each trial lasted approximately 30 s. Participants were instructed prior to each condition by means of verbal feedback given by the researcher and visual feedback presented on the screen. Participants were allowed to move the sliding foot panels prior to the start of the experiment, but the participants were not allowed to practice the different conditions.

In the haptic passive (HP) task, participants were asked to let their feet be moved passively while they rested their feet on the sliders. The feet were loosely strapped to the sliders to prevent slippage of the feet from the sliders and to prevent actions (active contributions to the motion) of the participant to compensate for any slipping. The participants were asked to look at a plus sign on the computer display before them while performing the haptic tracking task. The drivers, meanwhile, wore headphones and heard computer-generated auditory pacing sounds indicating the handle movements they were supposed to produce. As mentioned above, colored marks on the floor were provided to the drivers to help them control the amplitudes they were to generate.

In the haptic tracking (HT) task, participants were asked to actively track the motions of the sliders based on touch. Here, the definition of touch was relative, meaning that participants were instructed to feel the motion (e.g., amplitude and velocity) of the foot panels and move along with this motion. As in the HP condition, the subjects were asked to look at the plus sign on the computer display while moving their feet back and forth along with the guidance of the drivers. The difference between HP and HT lay in the instruction to the participants and the application of the straps in the HP condition. In all other respects, the procedure was the same as in the HP condition.

Note that the haptic tracking condition is not an exact copy of the haptic tracking condition in Rosenbaum et al. (2006), because of restrictions associated with cyclical movements of the feet compared to movement of the hands. In Rosenbaum’s study, the participant’s arms and hands were held in the air, touching a contact point moving on a frontoparallel surface, whereas in our haptic tracking condition participants' feet rested on sliding foot supports and participants were instructed to actively take part in the movement of the sliders. In Rosenbaum’s study, a technical precaution prevented the participant from generating enough force to allow for passive movement of the hands by the apparatus. Here, by contrast, we measured the forces which the participants generated horizontally and vertically and inferred from those forces how likely it was that the feet were being driven passively. Finally, in Rosenbaum’s study the coordination complexity was defined in terms of movement shape or movement frequency whereas here amplitude differences were targeted.

**Fig. 2:**
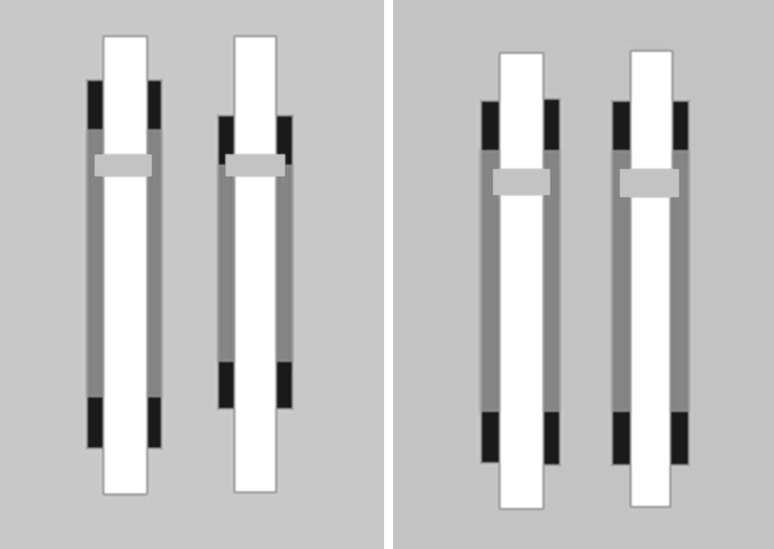
Visual feedback presented to the participants in the visual veridical (VV) task (*left panel*) and visual-enhanced (VE) task (*right panel*). The *green bars* moved up and down in concert with the out and in motions of the feet, respectively. The *left green bar* corresponded to the *left* foot, while the *right green bar* corresponded to the *right* foot. The different distances between the *dark blue targets* in the *left panel* reflect the shorter required amplitude for the *right* foot than the *left* foot. The equal distances between the *dark blue targets* in the *right panel* were shown no matter which foot had a longer or shorter amplitude (color figure online)

In the visual veridical (VV) task, participants were asked to move their feet back and forth to move a green bar between two dark-blue targets at the ends of a rectangle on the left while simultaneously moving a green bar between two dark-blue targets at the ends of a rectangle on the right. No drivers assisted with the motion in this condition. The visual feedback was veridical in the sense that the positions of the items on the screen corresponded directly to the required slider motions. Therefore, if, as shown in the left panel of Fig. 2, the right foot was supposed to cover 120 mm while the left foot was supposed to cover 150 mm, the lengths of the blue rectangles and the distances between the dark-blue targets corresponded to those values. Participants were instructed to move as quickly and accurately as possible in this condition. In all other respects, the procedure and design were the same as in the previously described conditions.

Finally, in the visual-enhanced (VE) condition, the procedure was the same as in the VV task except that bars of equal length were always presented, even in trials in which an amplitude difference was required. The gain of the system mapping the motions of the foot supports to the depicted target motions was adjusted, with, respectively, a factor 1.25 (120–150 mm) and 1.67 (90–150 mm), so that the targets would appear to cover equal amplitudes when the required slider amplitudes were produced. The right side of Fig. 2 gives an example. In all other respects, the procedure was the same as in the VV task.

### Data analyses

**Fig. 3:**
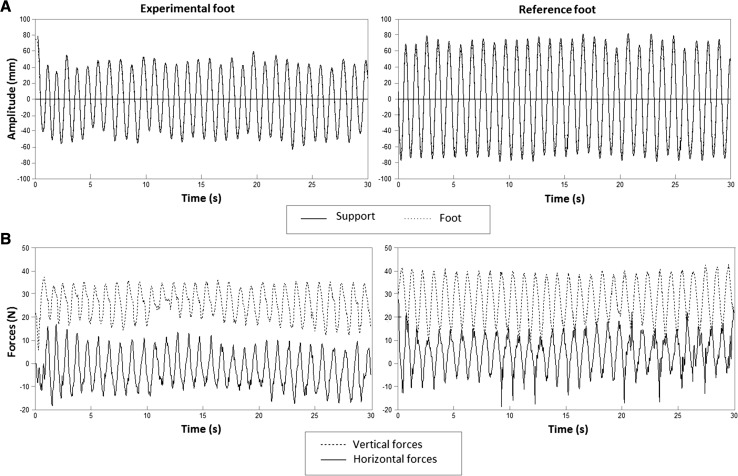
**a** Raw data time series of amplitude in mm produced by the experimental foot (*left panel*) and the reference foot (*right panel*) in the haptic tracking (HT) condition over 30 s. The *solid line* represents the amplitude of the foot support, and the *dotted line* represents the amplitude of the foot. *Solid* and *dotted lines* fully overlap since in this recording the movements of the feet and the supports corresponded maximally. **b** Raw data time series of the forces of experimental foot (*left panel*) and reference foot (*right panel*) exerted on the foot supports in a 30-s trial. The *dotted line* represents the vertical forces exerted on the foot supports, and the *solid line* represents the horizontal forces on the supports. The plots in **a** and **b** display data in a 60-mm amplitude difference condition (experimental foot: 90 mm, reference foot: 150 mm)

The position-time data were interpolated and filtered with a second-order, dual-pass Butterworth, low-pass filter with a cutoff frequency of 5 Hz. Subsequently, the signal’s offset was determined by calculating the signal’s average value, which was then subtracted from the signal. On the basis of a dedicated zero-crossing search algorithm, successive cycles were extracted. The first and last cycles of the trial were excluded from the analysis. For each obtained movement cycle, the realized amplitude, expressed in mm in the in–out dimension, was calculated to give the mean realized amplitude per 30-s trial. To control for potential slips, the correspondence between the movements of the foot support and the foot was calculated by subtracting the sum of the squared position-time curve of the IRED signal on the foot from that of the foot support and subtracting the result from 100 %. When the displacement of the foot was equal to the displacement of the foot support, the output was 100 %, reflecting that slipping of the foot from the foot support did not occur. In Fig. 3a, the raw position-time data of foot and foot support during a 30-s trial in the haptic tracking condition are presented.

To corroborate the hypothesis that haptic tracking facilitates the decoupling of the feet further, the correlations and continuous relative phase between the sagittal-plane position-time data of both feet were determined. The relative phase (mean and SD) was determined in degrees and was calculated using Batschelet’s (1981) procedures involving circular statistics (Meulenbroek et al. 1998).

The force data were filtered with a third-order, dual-pass Butterworth filter. The high-pass frequency for these signals was 0.1 Hz, and the low-pass cutoff frequency was set to 5 Hz. A peak detection procedure, written in MATLAB (R2012b), was used to determine the maximum positive force per movement cycle over the trial period of 30 s. For each trial, the median of the positive peak forces was calculated across all cycles. Figure 3b represents a 30-s trial example of the raw force signals in the haptic tracking condition.

### Statistical analyses

The mean realized amplitude (normally distributed according to the Kolmogorov–Smirnov test for normal distribution) was submitted to a repeated-measures analysis of variance (4 × 3 ANOVA) with the within-subject factors being condition (HP, HT, VV, and VE) and amplitude combination (150–150, 150–120, 150–90 mm). Using foot assignment as a between-subject factor in the repeated-measures ANOVA did not reveal an effect for foot assignment, and therefore, foot assignment was left out of further statistical evaluation.

The control variables: Correlations of the position-time data between the feet (Z-transformed) and the SD of the relative phase were also tested with a 4 × 3 repeated-measures ANOVA with condition and amplitude combination as within-subject-factors. To test for differences between HP and HT in correspondence between foot and foot support, pooled arcsine transformed fractions of the correspondence of HP and HT were submitted to a paired *t* test.

Median vertical and horizontal phasic forces proved also to be normally distributed and thus were submitted to a 4 × 3 repeated-measures ANOVA with condition and amplitude combination as within-subject factors. In case the assumption of sphericity was violated, the *F* value was adjusted using the Greenhouse–Geisser algorithm. The statistical analyses were conducted using SPSS statistical software, with alpha set to 0.05. If significance was found, post hoc comparisons of condition means were made to evaluate differences between the four conditions and the three amplitude combinations, and a Bonferroni correction was used to reduce the chance of type I errors. We also tested practice effects within the ten trials of each condition but did not find relevant main effects or interactions and thus pooled all analyses across this factor.

## Results

### Correspondence

The mean of the squared position-time signals of the sliding foot expressed as a percentage of the sliding foot supports exceeded 99 %, indicating that slippage of the feet from the foot panels was negligible. Correspondence in the HP condition was marginally but statistically significantly larger than in the HT condition (mean 99.94 % in HP versus mean 99.90 % in HT; *t*(16) = 7.36; *p* < 0.001).

### Amplitudes

**Fig. 4:**
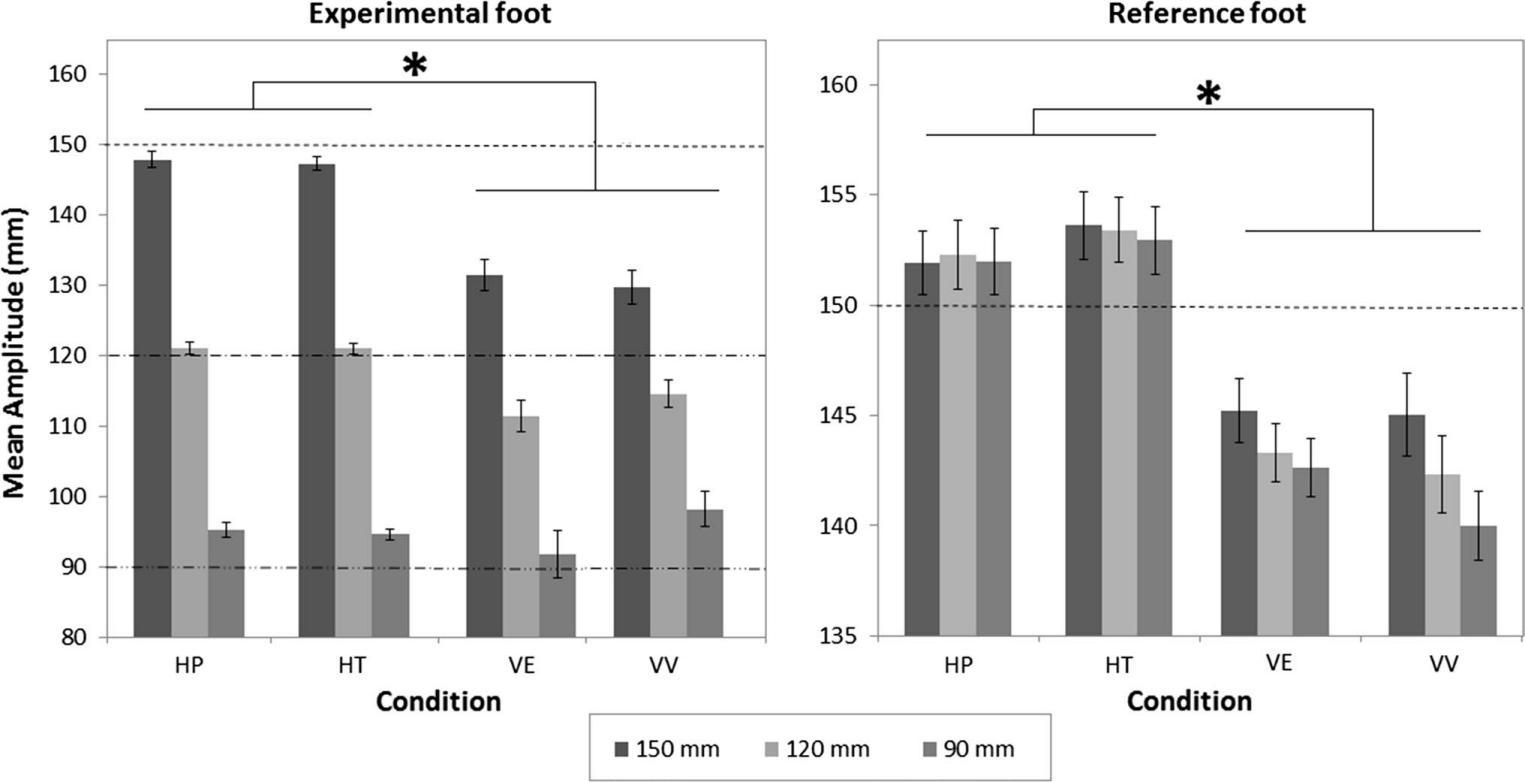
Mean realized amplitudes in mm produced by the experimental foot (*left panel*) and the reference foot (*right panel*) for the different condition–amplitude combinations. The legend indicates the requested amplitude of the experimental foot (also presented by the *dashed lines* in *left panel*). The target amplitude of the reference foot is 150 mm in all condition–amplitude combinations (also presented by the *dashed line* in *right panel*). *Error bars* represent ±1 SE, and *asterisk* indicates a significant difference between conditions, with *p* < 0.05. Note that the ordinates in the *left* and *right panels* are scaled differently

The realized foot amplitudes are shown in Fig. 4. The amplitudes for the experimental foot roughly approximated the required amplitudes. However, the amplitudes tended toward a middle value, showing an undershoot in the 150 mm condition and an overshoot in the 90 mm condition. This tendency was larger in the two visual conditions than in the haptic conditions, except in the VV condition in the 90 mm case. A repeated-measures ANOVA confirmed the significant interaction between condition and amplitude combination, *F*(6,96) = 32.10, *p* < 0.001. Post hoc comparisons with Bonferroni correction confirmed a significant difference between the haptic conditions and the visual conditions in the experimental foot (*p* < 0.001), indicating that the participants reached target amplitude better in the HP and HT conditions than in VV and VE (tendency toward the middle value was smaller in the haptic conditions).

As seen in the right panel of Fig. 4, with the reference foot subjects tended to overshoot in the haptic conditions and to undershoot in the visual conditions. Of greater interest, there was an effect of the experimental foot’s required amplitude on the reference foot in the visual conditions, but there was no such assimilation effect of the other foot’s required amplitude on the reference foot in the haptic conditions. As concerns the two haptic conditions, there was no difference in the amplitudes, but as concerns the two visual conditions, there was. A repeated-measures ANOVA confirmed the significant interaction between condition and amplitude combination, *F*(6,96) = 8.13, *p* < 0.001. In addition, post hoc comparisons, Bonferroni corrected, showed a significant difference between the haptic (HT and HP) and visual conditions (VV and VE) for the reference foot (*p* < 0.001). However, there was no significant difference between VV and VE.

**Fig. 5:**
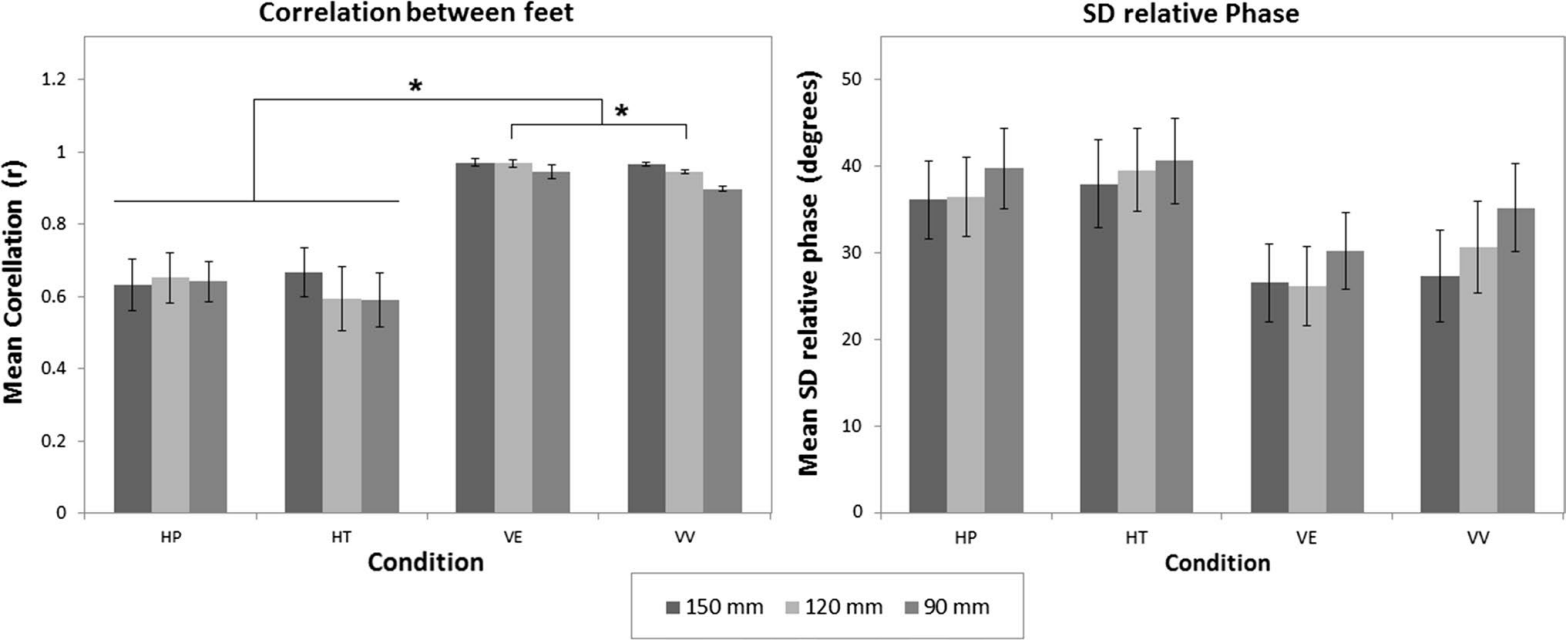
The *left panel* represents the mean correlations of the time-position signal between the feet for the different condition–amplitude combinations. In the *right panel*, the mean SD of the relative phase (in degrees) between the feet in the different condition–amplitude combinations is displayed. The requested amplitude of the experimental foot is shown in the legend. *Error bars* represent ±1 SE, and *asterisk* indicates a significant difference between conditions, with *p* < 0.05

The left panel of Fig. 5 shows the mean correlation between the position-time signals of the feet. The mean correlation between the feet was lower in the haptic conditions (*r* = 0.63) than in the visual conditions (*r* = 0.95). The repeated-measures ANOVA on the Z-transformed correlation coefficients revealed a significant interaction *F*(6,96) = 11.35, *p* < 0.001 between the two variables. Bonferroni-corrected comparisons confirmed a significant difference between the haptic conditions and the visual conditions (*p* < 0.001) and between VE and VV (*p* = 0.033).

The right panel of Fig. 5 shows that the SD of the relative phase was higher in the haptic than in the visual conditions. The repeated-measures ANOVA revealed a significant effect for amplitude *F*(2,32) = 17.44, *p* < 0.001 and an effect for condition *F*(3,48) = 2.79, *p* = 0.050 that just failed to reach statistical significance. In addition, no interaction was found.

### Forces

We focus next on the phasic forces that our subjects produced because we wanted to check that passive motion (HP) differed from haptic tracking (HT) in that HT entailed actively generated phasic foot forces, whereas HP entailed resting the feet on the sliders, having them act as ‘dead weights.’

**Fig. 6:**
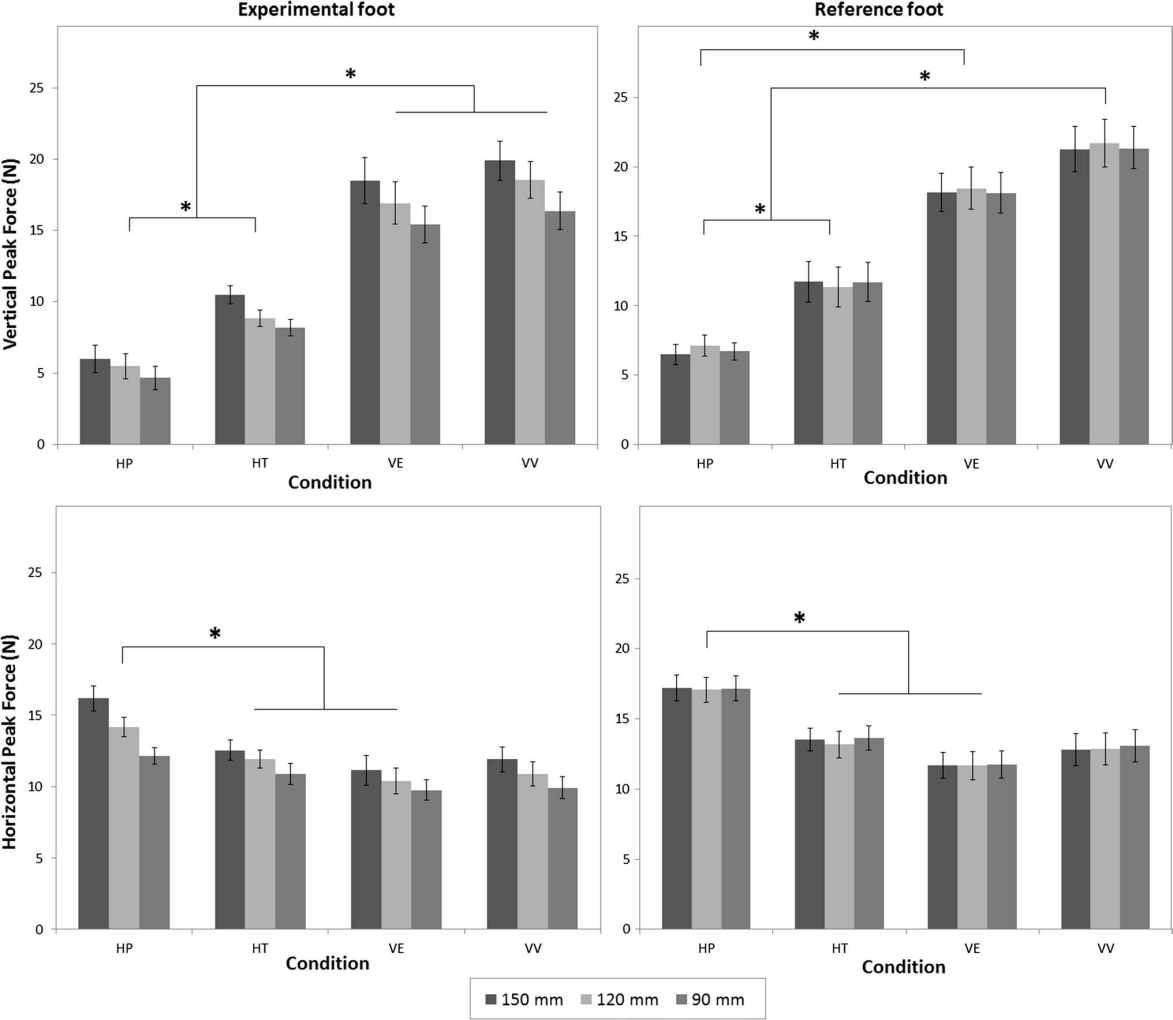
Group means of the median vertical and horizontal peak forces (Newton) exerted on foot supports in the four conditions and amplitude combinations. Vertical peak forces exerted on the sliding foot supports by the participant are presented in the *upper panels*. The *upper left panel* shows vertical peak forces for the experimental foot, and the *right upper panel* shows vertical forces applied to the sliding foot support of the reference foot. The *lower panels* show horizontal peak forces between foot supports and driver handles for the experimental foot (*left lower panel*) and the reference foot (*right lower panel*). The legend indicates the requested amplitude of the experimental foot, *error bars* represent ±1 SE, and *asterisk* indicates a significant difference between tasks with a *p* < 0.05

Figure 6, upper left panel, shows the median peak forces that subjects vertically exerted on the sliding supports with the experimental foot. As expected, the peak forces in the HP condition were lower than in the HT condition. In addition, the peak forces were substantially lower in the haptic conditions than in the visual conditions. We also found an effect of the required experimental amplitude. The median peak forces decreased with smaller amplitudes. When the experimental foot had to generate the smallest amplitude, the lowest force was exerted on the sliding support. These findings were backed up with a repeated-measures ANOVA, which revealed a significant interaction between condition and amplitude combination, *F*(6,96) = 4,55, *p* < 0.001. Bonferroni-corrected post hoc comparisons confirmed the differences between HP and HT and the difference between the haptic conditions and the visual conditions (VV and VE), as well as the differences between requested amplitudes (*p* < 0.01).

The upper right panel of Fig. 6 shows that the median vertical peak forces of the reference foot were significantly higher in the visual tasks than in the haptic tasks, and the peak forces in HP were again lower than the forces in HT. There was no effect of the experimental foot’s amplitude on the median peak forces of the reference foot. Repeated-measures ANOVA revealed a significant effect for condition, *F*(3,48) = 25.13, *p* < 0.001, but no interaction between condition and amplitude combination. Bonferroni-corrected comparisons of the four conditions again confirmed the differences between HP and HT and between the haptic conditions and the visual conditions (*p* < 0.01).

The lower left panel of Fig. 6 shows that for the experimental foot, we indeed found higher forces in the HP condition compared to the HT condition and the visual conditions. Furthermore, we found an effect of the requested amplitude on the amount of force applied to the sliders in horizontal direction. Similar to the vertical forces, this effect yielded a decrease in the peak forces applied on the sliders when smaller amplitudes were requested. Repeated-measures ANOVA confirmed the significant interaction between condition and amplitude combination, *F*(6,96) = 5.43, *p* < 0.01. Post hoc comparisons with Bonferroni correction confirmed that the difference between HP and the other conditions (HT and VV) was significant (*p* < 0.05), except for the difference between HP and VE (*p* = 0.059).

For the reference foot, as shown in Fig. 6 lower right panel, a significant effect was found for condition in horizontal peak forces, *F*(3,48) = 7.16, *p* < 0.01. The peak forces in the HP condition were significantly higher than in the HT and the VE conditions (*p* < 0.01, Bonferroni corrected). There was no significant interaction between condition and amplitude combination, and the requested amplitude of the experimental foot had no effect on the horizontal forces applied on the sliding foot support by the reference foot.

## Discussion

In this study, we asked whether bipedal coordination involving left and right foot displacements of different amplitudes would benefit from special haptic and visual feedback. Even though the haptic tracking paradigm for hand movements used by Rosenbaum et al. (2006) differed strongly from the haptic tracking paradigm for the feet used here, we found the same kind of benefits. The effects of visual feedback, however, were marginal. We first discuss the results with respect to the two modalities and then turn to broader issues.

### Haptic tracking

We hypothesized that if bipedal haptic tracking actually consists of guided movement (active generation of the feet’s movements) and not passive dragging, the phasic downward forces of the feet on the sliders would be significantly lower in the HP condition than in the HT condition. Consistent with this prediction, we found that the active contribution to the sliding in–out movements of the feet did indeed result in significantly lower phasic vertical forces on the foot plates in the HP condition than in the HT condition. In further support of the difference between passive movement and haptic tracking, the phasic horizontal forces on the foot sliders were lower during haptic tracking than during passive movement, again resulting from an active phasic contribution during haptic tracking. In the HT condition, participants helped the drivers generate the movements of the sliders by generating forces in the same in–out direction, resulting in lower net forces. By contrast, in the passive condition the participants let their feet dragged by the drivers, who as a result had to produce higher phasic forces.

Given that haptic tracking occurred, we can turn to the core hypothesis of our study. Our results confirmed that the reference foot was less affected by the experimental foot in the haptic conditions (HP and HT) than in the visual conditions (VV and VE). In other words, haptic tracking facilitated uncoupling of the feet’s preferred mode to generate identical amplitudes. As expected, both the experimental foot and the reference foot approached target amplitude significantly better in the haptic conditions, with only marginal deviations from target amplitude (<5 mm), whereas in the visual conditions the feet showed deviations up to 20 mm from the target amplitude. Without haptic guidance, participants generated relatively large undershoots. In addition, the inter-pedal correlations and the standard deviations of the continuous relative phase between the feet movements confirmed that the uncoupling of the movements of the feet was more pronounced in the haptic than in the visual conditions. The correlation coefficient approximated 1.0 in the visual conditions, implying a strong coupling of the feet. In the haptic conditions, the correlation coefficient was significantly lower (*r* = 0.6), which shows that a substantial degree of uncoupling of the feet occurred. Taken together, bipedal coordination involving different amplitudes benefited from haptic tracking, consistent with the hypothesis that haptic tracking reduced the motion planning challenges of moving the two feet at different amplitudes.

### Enhanced visual feedback

As haptic tracking enhanced inter-limb coordination, we expected to find similar beneficial effects of enhanced visual feedback compared to veridical visual feedback. However, we failed to find a significant difference between the realized amplitudes when participant was provided with normal, veridical feedback (VV) or enhanced, simplified feedback (VE). In both visual conditions, the realized amplitudes of the reference foot were clearly affected by the amplitudes of the experimental foot, and participants even reported that they perceived the condition with enhanced visual feedback to be more difficult than the condition with veridical visual feedback. However, the inter-pedal correlations differed significantly between VV and VE: The correlations between the feet were affected by a difference in amplitude in the VV condition, but not in the VE condition.

Our results concerning the use of simplified visual feedback to enhance inter-limb coordination are at odds with the literature on bimanual coordination, showing that complex bimanual movements are performed with greater ease when the task is presented as a simple unified task or when augmented online visual feedback is provided (e.g., Boyles et al. 2012; Diedrichsen et al. 2001; Franz and McCormick 2010; Franz et al. 2001; Kovacs et al. 2010; Mechsner et al. 2001; Wang et al. 2013). This difference might be attributed to a difference between this study and others with respect to the length of training. The participants in Mechsner's experiment were given 20 min of practice before the start of the experimental trials. Other researchers, using Lissajous visual feedback to help subjects bimanually perform a 2:1 frequency ratio or 90° phase lags, have even permitted participants several days of practice (Summers et al. 2002; Swinnen et al. 1997). In our experiment, by contrast, participants immediately started with the experimental trials.

Another possible reason why we failed to find significant differences between the normal, veridical feedback and the enhanced, simplified feedback conditions might be related to the relative ease of our tasks. In the complex task that Mechsner et al. (2001) used, participants had to produce a difficult 4:3 bimanual polyrhythm. Others exploring the effects of enhanced visual feedback have used different phase relations that, without enhanced visual feedback, are very difficult to produce (Bogaerts et al. 2003; Kovacs et al. 2010; Mechsner et al. 2001; Wilson et al. 2010). Our task, in which we requested different amplitudes of the feet, may not have been that hard. This might have been reflected in the fact that even in the visual veridical condition of our experiment, participants produced target amplitudes with deviations of just 10 mm in the control foot and 20 mm in the experimental foot, i.e., ranging between 6.7 and 13 % of the target amplitudes, respectively. However, in pilot tests we found phase and frequency differences were too difficult for subjects in the context of our bipedal tasks, and we reasoned that amplitude differences combined with in-phase foot movements (whereas feet usually move in anti-phase) would be sufficiently complex to be able to test the differential effects of haptic versus visual feedback.

Another explanation might be found in the fact that some of the studies mentioned above used a unified presentation of the task to be executed: The two separate task components representing the two distinct actions of the hands were reduced to a single representation. Here, two rectangles represented the two separate target amplitudes that had to be reached by the participant’s feet on the sliders. Consequently, the cognitive load that our subjects experienced might have been roughly equivalent in the same and different amplitude conditions where visual feedback was provided, either in veridical (VV) or simplified form (VE).

Finally, the absence of an effect for enhanced visual feedback might have derived from the fact that the visual feedback we provided was presented on a screen in front of the participant, and so in a different plane than the one in which they moved. Participants received feedback of the task features in the frontoparallel plane, whereas the actual movements they made with their feet were in the horizontal plane. In planning the foot movements, the participants had to transform the visual feedback from the frontoparallel plane to execution in the horizontal plane. This internal transformation may have diluted the benefit that could have accrued from the simplified visual feedback.

Although we hypothesized that bipedal coordination would benefit from enhanced visual feedback and we searched for different explanations why no benefits were found in the present study, we wish to mention that our results are actually in line with some previous work on bimanual coordination. Weigelt and Cardoso de Oliveira (2003) also used visuomotor transformations in making bimanual discrete movements with different amplitude combinations and found that bimanual movements with different amplitudes were always accompanied by an assimilation effect: even when movements appeared to be of the same amplitude on the screen. These authors concluded that movement assimilation was not affected by visual feedback, but only by the executed amplitudes. However, they found that transforming visual feedback did affect other measures of bimanual coupling such as the bimanual amplitude correlation. We found a similar effect for bipedal correlations.

### Limitations

In addition to the issues mentioned above, this work had some limitations. First, we used human drivers, partly out of expediency. Using one or more robots or other electromechanical systems might have afforded greater precision. Nevertheless, our post hoc analyses showed that the mean and variability of the frequency (in Hz) of the sliding in–out movements was quite on target (1 Hz) and stable in the haptic tracking condition (*M* = 1.05 Hz, SD = 0.03 Hz), compared to the VE (*M* = 0.71 Hz, SD = 0.20 Hz) and VV condition (*M* = 0.75 Hz, SD = 0.21). Moreover, our human–driver approach, besides being similar to what was done in the earlier haptic tracking study, is comparable to what happens in typical clinical contexts. Had we used a mechanical system to drive the movements, the question would have remained as to whether in typical clinical contexts human drivers (therapists) could successfully administer haptic tracking. That question has been answered here in the affirmative.

A second limitation of this study was related to our decision to use straps to prevent the feet from slipping in the haptic passive task. This might be considered a confound in the comparisons of the effects of the two haptic conditions, for we did not apply straps to the feet in the active haptic tracking condition, nor did we do so in the two visual conditions. As stated above, however, we used the straps in the haptic passive task to help our participants stay on the rails and prevent them from generating compensatory actions to prevent slippage. Even so, we were concerned about the fact that straps were used only in one condition and sought to investigate this matter further. We separately analyzed, by means of a low-pass filtering technique, the *constant* forces applied to the sliding foot supports to maintain contact (frequency <0.1 Hz). We found no differences between the haptic passive and haptic tracking condition in this analysis, adding to our confidence that the straps did not cause a measurable confound.

A third limitation of our study was that we used a single reference amplitude of 150 mm, which was paired with experimental amplitudes of 150, 120 or 90 mm. It is possible that the influence of the experimental amplitude on the reference amplitude, and the interaction of this factor with condition (HP, HT, VV, or VE) might have depended on which reference value was used. By assigning the largest amplitude to the reference amplitude, we could observe the effects on the reference amplitude of as large an amplitude difference as we thought we could use, namely a 60-mm difference. It is an open question what would happen if other pairs of left and right foot amplitudes were used.

### Clinical implications

As we stated in the introduction, we were partly interested in studying bipedal coordination because we thought enhanced visual feedback and haptic tracking might prove to be useful for rehabilitation. We failed to find benefits of enhanced visual feedback on bipedal coordination, but we did find benefits of haptic tracking for the movements of the two feet. This is the first time haptic tracking has been used to see whether this method can be used to facilitate spatially incongruent foot movements. We have shown that it can be.

The efficacy of haptic tracking has potentially important clinical implications. Haptic tracking can be used as a rehabilitation tool to bypass motor planning or control difficulties that some patients have. By leading patients in the way that haptic tracking permits, patients can be enabled to exhibit a wider behavioral repertoire than they might show otherwise. This might prove especially valuable in situations where patients persist in producing adaptive behavior that was appropriate for the duration of a musculoskeletal injury but is maladaptive after healing has occurred (Walter and Swinnen 1994). Successful task completion with haptic tracking may also boost motivation (Marchal-Crespo and Reinkensmeyer 2009).

Haptic tracking bears a resemblance to active-assistive motion, a technique that is often used in physical rehabilitation, where the physical therapist and patient engage in joint action in which the physical therapist leads and the patient follows. The leader is responsible for guiding the joint action and initiating transitions through different movement sequences, thereby allowing the joint action to be smoothly coordinated. Active-assistive motion therapy is used, for example, to increase the range of shoulder motion (Goldberg et al. 2001; Uhl et al. 2010) or promote full extension and flexion of the knee after repair of the anterior cruciate ligament (De Carlo and McDivitt 2006; Noyes et al. 1992). Insofar as active-assistive motion may in fact comprise a mixture of passive movement and haptic tracking, it is important to know that haptic tracking per se can be beneficial. Our results suggest that haptic tracking may provide a new and potentially important treatment option to help patients regain lost motor capabilities of the lower extremities.
